# Taking Stock: Elite Studies and Social Change

**DOI:** 10.1111/1468-4446.70093

**Published:** 2026-04-07

**Authors:** Lena Ajdacic, François Schoenberger, Johanna Behr, Fabio Cescon, Lion Hubrich, Emma Ischinsky, Muneeb Salman, Juliana Mazzucotelli, Laura Mrčela, Alonso Pi Cholula, Céline Roy, Sophie Serrano, Helle Dyrendahl Staven, Tina Thakur

**Affiliations:** ^1^ University of Lausanne Lausanne Switzerland; ^2^ University of Oxford Oxford UK; ^3^ Centre Marc Bloch Berlin Germany; ^4^ University of Amsterdam Amsterdam the Netherlands; ^5^ Humboldt‐Universität zu Berlin Berlin Germany; ^6^ MPIfG Cologne Germany; ^7^ National Defence University Islamabad Pakistan; ^8^ University Grenoble Alpes Saint‐Martin‐d'Hères France; ^9^ University of Ljubljana Ljubljana Slovenia; ^10^ El Colegio de México Mexico City Mexico; ^11^ University of Neuchâtel Neuchâtel Switzerland; ^12^ Oslo Metropolitan University Oslo Norway; ^13^ National Institute of Educational Planning and Administration New Delhi India

**Keywords:** elites, power, review, social change

## Abstract

This article provides a systematic synthesis of contemporary elite sociology through the analytical lens of change and stability. We distinguish between two types of change: change within elites, referring to transformations in elite composition, circulation, or internal characteristics; and change by elites, designating processes whereby elites reshape broader social structures, norms, or inequalities. Through a systematic review of 164 empirical articles published in leading sociology journals between 2000 and 2024, we demonstrate that contemporary elite sociology engages extensively with temporal perspectives, though in asymmetric ways. By systematically examining combinations of elite types and research topics, we reveal systematic patterns: studies addressing nationality emphasize evolution and shifting configurations, while research on kinship and class more frequently examines continuity and reproduction. Crucially, we find that broader social change associated with elites is predominantly portrayed as an unintentional consequence rather than the result of deliberate strategic action. Based on these findings, we outline four directions for future research: reversing the temporal lens to investigate understudied dynamics; situating elite processes along a temporal spectrum that captures both continuity and transformation; examining the degree of intentionality in elites as agents of change; and integrating forward‐looking perspectives to understand how elites imagine and actively shape trajectories. This synthesis advances elite sociology by revealing how temporal perspectives fundamentally structure our understanding of power relations and by identifying critical gaps in how we conceptualize elites' relationship to social transformation.

## 1 Introduction

Recent influential scholarships argue that elite hierarchies continue to reproduce themselves across generations. The same families, schools, and institutions persist in shaping access to positions of power, resulting in remarkable continuity in elite composition (Reeves and Friedman 2024). Simultaneously, scholars contend that the expansion of elites contributes to broader social transformations, such as the erosion of trust in state institutions and the decrease of civic cohesion (Turchin 2023). An underlying question in these studies is the tension between change and stability. This consideration is crucial because it directly reflects whether and how the distribution of power in societies is reproduced or challenged, and, more generally, how social orders are maintained, contested, and transformed.

For early elite scholars, broad theories of social change were central. In 1916, Vilfredo Pareto used the analogy of a river and the flow of water to illustrate the constant dynamic in the ruling class. In the post‐war era, scholars turned their attention to the role of elite replacement in enabling democratic stability (Dahl 1961; Field and Higley 1980; Keller 2017). Critical elite scholars, in turn, highlighted how the growing power of the military and business sectors reshaped societies (Mills 1956). Building upon these foundational works, recent calls to contemporary scholars have encouraged greater attention to the capacity of elites to evolve (Cousin et al. 2018). However, fundamental questions remain to be explored: are elites themselves understood as evolving entities? Are they discussed as impeding or enhancing broader social change? This article addresses these gaps.

Our article provides a synthesis of contemporary elite research through the analytical lens of change and stability. Previous agenda‐setting articles have provided overviews and pathways for the development of elite sociology based on the knowledge of leading scholars (see Bukodi and Goldthorpe 2021; Cousin et al. 2018; Davis and Williams 2017; Khan 2012; Korsnes et al. 2017). We advance these perspectives through a systematic review of what has become a rapidly expanding field. We systematically examine a corpus of 164 key publications in the sociology of elites published between 2000 and 2024. When scholars clearly state they are examining elites we take an interest in their work, as research on elite dynamics requires understanding the contexts in which elites are selected and trained, the groups that aspire to or challenge elite power, and the broader legitimization strategies and shared perspectives that underpin elite influence.

First, we are interested in whether elite literature emphasizes change or stability. Second, for articles that adopt a temporal perspective, we distinguish between two types of change: change *within* elites and change *by* elites. In theoretical terms, different kinds of change can be distinguished depending on which aspects and dimensions of a system are involved (Sztompka 1994). Change *within* elites refers to transformations in the elite itself: changes in composition, circulation, attitudes, or internal characteristics of elite members, regardless of their effects on broader society. Change *by* elites designates processes whereby elites are agents of transformation in society at large: altering norms, policies, institutional arrangements, or patterns of inequality. The distinction highlights that although a central claim in elite sociology is that elites are the groups exercising influence, relatively few studies have empirically examined how such influence is exerted.

Our analyses show first how scholars discuss continuities and shifts *within* elites. We identify which types of elites—such as business, political, cultural, and wealth elites—and which research topics are approached through the lens of stability or change. Using a “matrix of change,” we demonstrate that topics such as “Nationality” highlight shifts and evolution, whereas topics such as “Kinship” and “Class” are more often examined in terms of continuity. Second, we show that fewer studies address change driven *by* elites, but the spheres of influence explored are highly diverse—for instance, political elites stabilizing foreign policy, cultural elites reshaping Black cultural production, or wealth elites influencing anti‐democratic attitudes. A key insight from this analysis is that broader social change associated with elites is frequently portrayed as an unintentional consequence of their actions.

Based on this review, we outline four directions for future research. First, reversing the usual temporal lens employed to investigate elites and focus on understudied dynamics. Second, situating observations along a temporal spectrum that captures both continuity and transformation. Third, examining the degree of intentionality in elites as agents of change. Fourth, integrating visions of the future into elite studies to better understand how elites imagine what is to come.

The focus on social change is crucial, as temporal perspectives inherently shape how power relations are understood. Viewing elites through the lens of stability tends to depict power relations as static, portraying elites as a group that only fosters inertia, resists renewal, and sustains existing social structures. Conversely, examining elites through the lens of change emphasizes the evolving and adaptive nature of power relations. Our study brings not only conceptual clarity but also shifts attention on elites and social change in a context of ongoing contestations surrounding democracy's role in contemporary societies.

## 2 The Prism of Social Change in Elite Studies: A Systematic Review

### 2.1 Sampling Strategy

The systematic review is grounded in the collaborative work of 14 new generation scholars in elite research (details on the collective are provided in Appendix A1). We identified a *literature corpus* with a selective sample of works in sociology. It includes 164 empirical articles published between 2000 and 2024 in 20 leading sociology journals, featuring the term elite/s in the title.

Our analysis benefits from recognizing the diversity of elite definitions. An understanding of elite dynamics requires an understanding of the contexts in which elites are selected and trained, of those groups aspiring and challenging elite power and of the larger legitimization strategies that underpin elite influence. There have been efforts to clarify the boundaries of elite sociology, building on Khan's (2012, 362) definition of elites as “those with vastly disproportionate control over or access to a resource”. Attempts to standardize the definition of elites work toward operationalisable definitions at the level of individuals and organizations (Bühlmann et al. 2025). However, the existing body of literature in elite studies does not follow a unified conceptualization of elites.

In this study, we thus adopt an inclusive approach to the definition of elites by embracing the definitions used by authors themselves. If researchers claim to study elites, we take an interest in their work. With one exception: articles on sports elites were excluded, as their definition differs from the understanding of social power that guides our interest. To better understand which segments of elite research we might run under other terms than elite/s, we cross checked a list of terms that are related, for details see Appendix A2. It shows that our sampling approach likely underestimates the significance of work on income elites. Further discussions that our sampling approach might miss are situated in a Gramscian tradition, which operate with notions such as the ruling class or the international capitalist class. The focus enabled us, however, to work with a corpus that is manageable in size for manual reading and analyses of all articles.

To examine key publications, we selected articles published in 20 leading sociology journals, including top general sociology outlets and journals particularly relevant to elite research. For the full list of journals used for article selection, see Appendix A3. The corpus was restricted to empirical articles published in English, as this is the dominant language in the internationally oriented academic space. We exclusively included journal articles and excluded books and book chapters, to focus on peer‐reviewed research. Selecting only empirical studies allowed us to see how scholars discuss change and stability within specific geographic and temporal contexts. This selection process resulted in 164 articles. As shown in Appendix A4 for our literature corpus and Appendix A5 for a broader Web of Science sample, elite sociology, and elite studies more broadly, are growing fields.

### 2.2 The Coding Process

For the analysis, each article was systematically examined to understand how change is discussed theoretically and treated empirically. We employed a 25‐item review framework, analyzing all articles in the corpus across two key dimensions. First, we assessed each study's empirical coordinates, including geographic focus, elite type, research object, methods, and data structure. Second, we investigated how temporal dimensions were addressed, analyzing the factors driving change or stability, their consequences, methodological approaches, and the connections between temporalities and conceptions of power.

Each article was assigned to one elite type: “Business Elite” (including studies on top managers in corporations, or on specific subgroups in the economy such as executives, entrepreneurs or owners in finance or tech), “Education Elite” (including studies on elite universities or scientific elites), “Political Elite” (including studies on policy elites, parliamentarians, top level groups in state administrations), “Cultural Elite” (including studies on elites in culture and arts), “Wealth Elite” (including studies on both wealthy and high income individuals or families, and on philanthropy related subgroups), “Multiple Elites” (including studies that regroup any of the previous elite clusters). Most articles could be readily assigned to one of these categories. Only a few articles in our sample focused on more unusual elite subgroups such as juridical elites, the high‐status party scenes, or elites in military, which we grouped together under “Other Elites”.

To classify the temporal orientation of articles, we relied on common temporal indicators. Discussions of stability were typically evident when authors described how elites reproduce, maintain, persist, or engage in processes of closure, exclusion, and continuity. More technical terms such as constant, durable, steady, or unchanged also signaled stability. In contrast, discussions of change often referred to the rise or decline of elites, the emergence of new elites, or broader shifts and transformations. Technically, this was expressed through terms such as increase, decrease, growth, or gains. The advantage of researcher‐coded classifications over automated text processing, however, is our enhanced capacity to capture the complexity of argumentative contexts. In cases of temporal ambiguity, and whenever possible, we coded according to the more dominant orientation. In general, it was more straightforward to assign temporal orientation in theoretical sections. In empirical sections, for example, ambiguity arose when research relied on longitudinal data, addressed the evolution of a phenomenon, but treated trends only marginally because a comparative question was central to the study, or when the primary contribution was methodological one. Although our approach results in a simplified categorization, it enables us to identify general temporal orientations across different segments of the literature and to examine the blurring of temporal ideas more thoroughly.

To ensure coding consistency, 30% of the corpus was independently coded by two researchers. This intercoder reliability check allowed for the comparison of results and allowed us to resolve discrepancies and ensure alignment on coding. The review was carried out in three iterations, with plenary sessions and discussions in between to ensure a coherent approach across the members of the author collective.

### 2.3 Methods

To explore the articles discussing issues *within* elites, we created a matrix that displays the distribution of articles for elite type and research topics. The combination of those creates insights in what we call hyphenated elite research fields, such as the “Network ‐ Business Elite” sociology, or the “Gender—Wealth Elite” sociology. Each article was coded for one or more research topics. We applied a non‐exclusive coding scheme, as many articles address several themes simultaneously. The classification is organized into ten categories, designed to be internally coherent, as clearly distinct from one another as possible, and aligned with keyword usage commonly employed by researchers in the field. Coding was based on a detailed reading of the full articles. The categories are: Race (studies including discussions on race, ethnicity and skin color); Kinship (studies on family or inter‐generational links); Gender (studies on gender, masculinities or women); Class (studies discussing social origins or class structures); Distinction (studies on symbolic boundaries, delineation processes, status symbols); Access (studies on educational paths, careers, recruitment strategies, or professional pathways to top positions); Networks (studies on links between elites or organizations, processes of cohesion and closure or social capital); Nationality (studies on national, international, global issues regarding elites); Resources (studies addressing the role of monetized assets, income or institutional resources); and Attitudes (including studies on political views, opinions, or moral sentiments). These categories capture the most important research topics present in the literature corpus. Some topics that might be expected to play a larger role, such as religion, as well as sexuality, emotions, or spatiality, and migration appeared only marginally in the literature and were therefore not included in the definition of research topics.

To explore the articles discussing change driven *by* elites, we provide an overview on types of elites and the diversity of spheres of influence as discussed in the articles. We show the number of contributions by elite type. For each elite type (e.g., Business Elites, Political Elites, Wealth Elites, Cultural Elite), we identify a specific sphere of influence (e.g., employment relations, race‐based policies, cultural production). We defined these by a detailed reading of the full articles, assessing how authors discuss the influence of elites in each article. We examined how intentionality is attributed to elite action, focusing in particular on explicit references to intention, strategy, and agency. In addition, we analyzed the mechanisms linking elite behavior to observed outcomes, distinguishing between studies that discuss elite influence primarily at a theoretical level and those that investigate it empirically. Finally, we assessed whether articles frame elites more as guardians of the status quo, enhancing stability, or as agents of change.

To assess the temporal focus in both categories of articles, we worked with the temporal classification of articles' theory sections. The author collective assessed each article through several steps: (1) Does the article have an overall temporal focus; (2) Is there a temporal focus in the theory section; (3) Does it emphasize change or stability.

## 3 Social Change *Within*, or Social Change *By* Elites?

### 3.1 Literature Corpus Description

The sociology of elites includes a wide range of approaches. To provide orientation, we show descriptive analyses on the literature corpus (*n* = 164), presented in Table 1. In terms of methodology, 47.0% of the studies use quantitative methods, 40.9% employ qualitative approaches, and 12.2% adopt a mixed methods design. This methodological diversity suggests a shift away from the earlier reliance on survey data, which has been identified as a limitation in studying the dynamic nature of contemporary elites (Savage and Williams 2008). We note that qualitative and quantitative articles engage with social change in different ways. Quantitative studies typically examine trends, or the absence of trends, using longitudinal data and a wide range of methods, including network analyses, descriptive statistics, regression models, and event‐history approaches. Qualitative studies, in turn, situate empirical material within broader historical and social contexts. They draw on interviews and life stories that link individual narratives to political or social events, long‐term ethnographic research, and group discussions that encourage collective reflection on how communities perceive their own evolution. Such methods often illuminate both shared and contested understandings of change.

**TABLE 1:** Descriptives of the elite sociology literature corpus.

	*N*	%
Country studied		
US	46	28.1
UK	28	17.1
Multiple countries	22	13.4
Sweden	14	8.5
China	8	4.9
Norway	7	4.3
Finland	5	3.1
Switzerland	5	3.1
Czech Republic	4	2.4
Denmark	4	2.4
Germany	3	1.8
Brazil	2	1.2
Chile	2	1.2
Europe	2	1.2
France	2	1.2
Bolivia	1	0.6
Canada	1	0.6
Croatia	1	0.6
Israel	1	0.6
Japan	1	0.6
Madagascar	1	0.6
Nigeria	1	0.6
Singapore	1	0.6
South Africa	1	0.6
Spain	1	0.6
Method		
Quantitative	77	47.0
Qualitative	67	40.9
Mixed	20	12.2
Geographic scale		
National	119	72.6
International	19	11.6
Local	14	8.5
City	12	7.3
Key theory reference^a^		
Bourdieu	71	43.3
Mills	20	12.2
Khan	18	11.0
Domhoff	9	5.5
Tilly	3	1.8
Type of elite		
Business elite	44	26.8
Educational elite	30	18.3
Multiple elites	26	15.9
Political elite	24	14.6
Wealth elite	20	12.2
Cultural elite	12	7.3
Other elite	8	4.9

^a^ Multiple/no reference(s) possible.

Geographically, studies are concentrated on Western contexts, reflecting our linguistic selection criteria. Given the dominance of English in Western academic publishing, our anglophone corpus may underrepresent scholarship on non‐Western elites published in other languages. Studies focusing on elites in the United States or the United Kingdom account for 45.2% of the sample, followed by research on Sweden (8.5%) and China (4.9%). Most empirical studies focus on national elites (72.6%), while a smaller share examines international elites through cross‐national comparisons, global business networks, or transnational policy‐planning groups (11.6%). A further 8.5% explore elites at the local level, such as within universities, college campuses or neighborhoods, and 7.3% study elites in particular cities, such as in Oslo, Dallas, Helskinki or Dhaka.

In terms of theoretical orientation, 43.3% of all articles cite Pierre Bourdieu as one main theoretical reference, which might support the rationale behind recent calls for theoretical diversification (Cousin et al. 2018). Bourdieu's prominence is notable, especially since he referred to the “field of power” (see e.g. Bourdieu 1998) rather than explicitly using the term “elites.”

### 3.2 A Focus on Change

**FIGURE 1:**
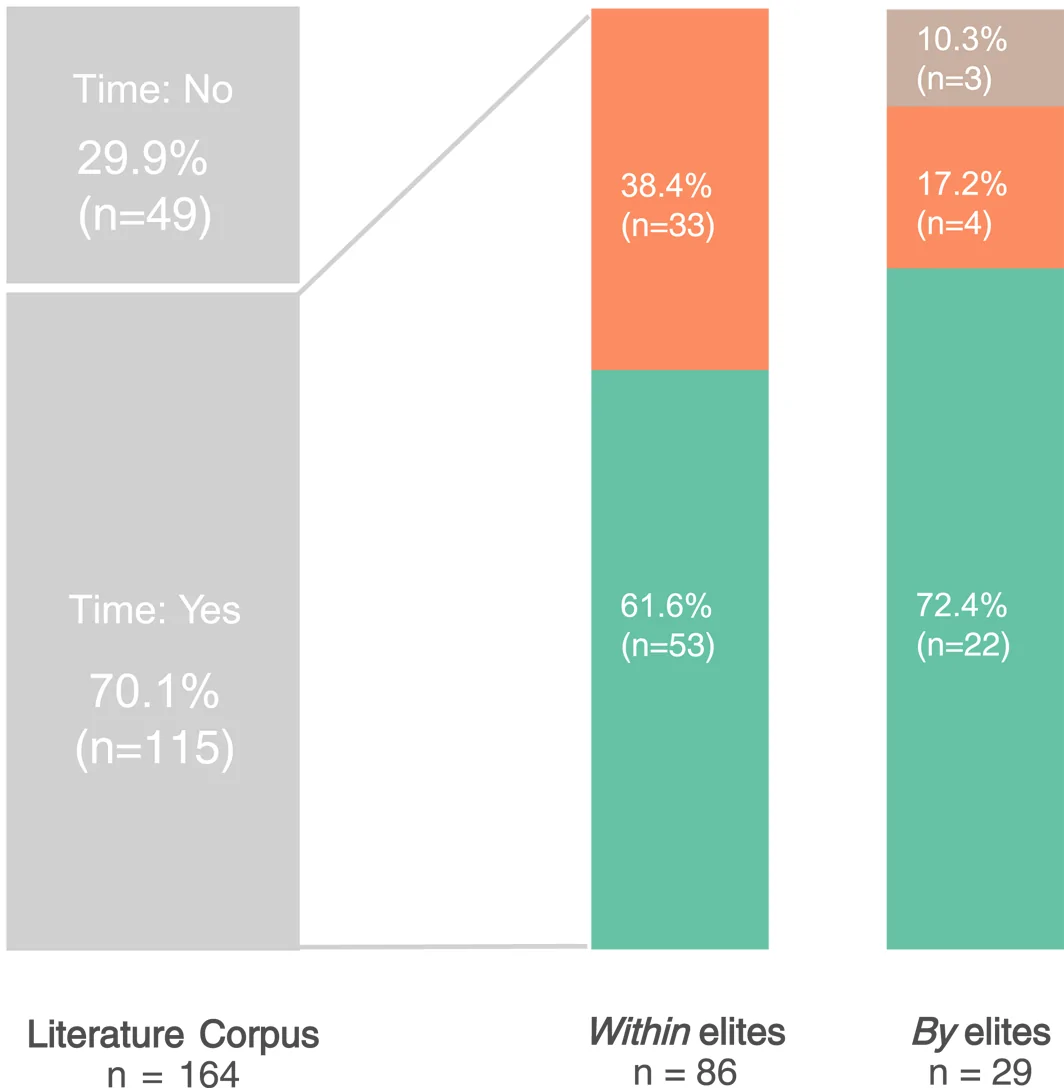
Temporal perspectives in the literature corpus. The bar plot shows the distribution of articles according to their temporal focus, or absence thereof. Colors indicate articles with no temporal dimension (gray), a focus on change (green), a focus on stability (orange), or both (brown), based on classifications from the theory section. The bars to the right show that among articles with a temporal focus, 86 focus on issues *within* elites, and 29 focus on issues *by* elites.

The distinction between studies that focus on *within* elite issues and studies that focus on issues influenced *by* elites form the basis for our in‐depth analysis of how concepts of social change are articulated in elite studies. Figure 1 presents the temporal focus of the articles. The classification is based on the theory section of the articles. While we find that the majority of articles do engage with temporal perspectives, 29.9% of publications do not address temporal perspectives at all. Among articles without temporal focus there are for example descriptive studies that examine networks, careers, cultural practices, or ideologies at a specific point in time, analyses of intra‐elite relations, cross‐national or group comparisons, and in‐depth examinations of specific mechanisms of inequality, exclusion, or legitimation.

Most contemporary articles, however, do engage with temporal perspectives. To analyze how scholars discuss change, or resistance to change, we distinguish between two groups of articles. The first, large group (*n* = 86) focuses on issues *within* elites. Studies which we group in this category examine internal elite dynamics, such as access to elite positions, career trajectories, or class and gender composition in different types of elites. Among them 61.6% focus on change (shown in green), while the remaining 38.4% focus on stability (shown in orange). This means that studies on issues *within* elites focus more on evolving aspects of elite access and composition, rather than on how elites resist change within their own ranks.

The second group (*n* = 29) and, interestingly, much smaller group of articles, discusses how social change or stability are influenced *by* elites. Studies which we group in this category investigate how elites shape social processes as agents or gatekeepers of transformations. Among them 75.9% focus on change, while the remaining third focuses on stability or addresses both dimensions.

This overview does not allow us to determine whether elites are indeed evolving more strongly or to identify which subtypes of elites might be doing so. Rather, it shows how authors approach the study of elites and highlights the main temporal focus in their conceptualization and discussion of elite actors. Thus, while there have been calls for a stronger focus on how elites change (Cousin et al. 2018), our analysis demonstrates that contemporary empirical elite sociology already considers social change as a central concern.

## 4 Continuities and Shifts *Within* Elites

This section considers how elites are portrayed as maintaining continuity by blocking access, preserving social closure, reproducing privilege, and sustaining the dominance of established groups. Or, in turn, how elites are discussed as evolving, for example through increasing openness, growing diversity, decreasing cohesion, and the rise of emerging actors. In this section, we focus on the 86 articles that address continuities and shifts *within* elites.

### 4.1 The Matrix of Change

**FIGURE 2:**
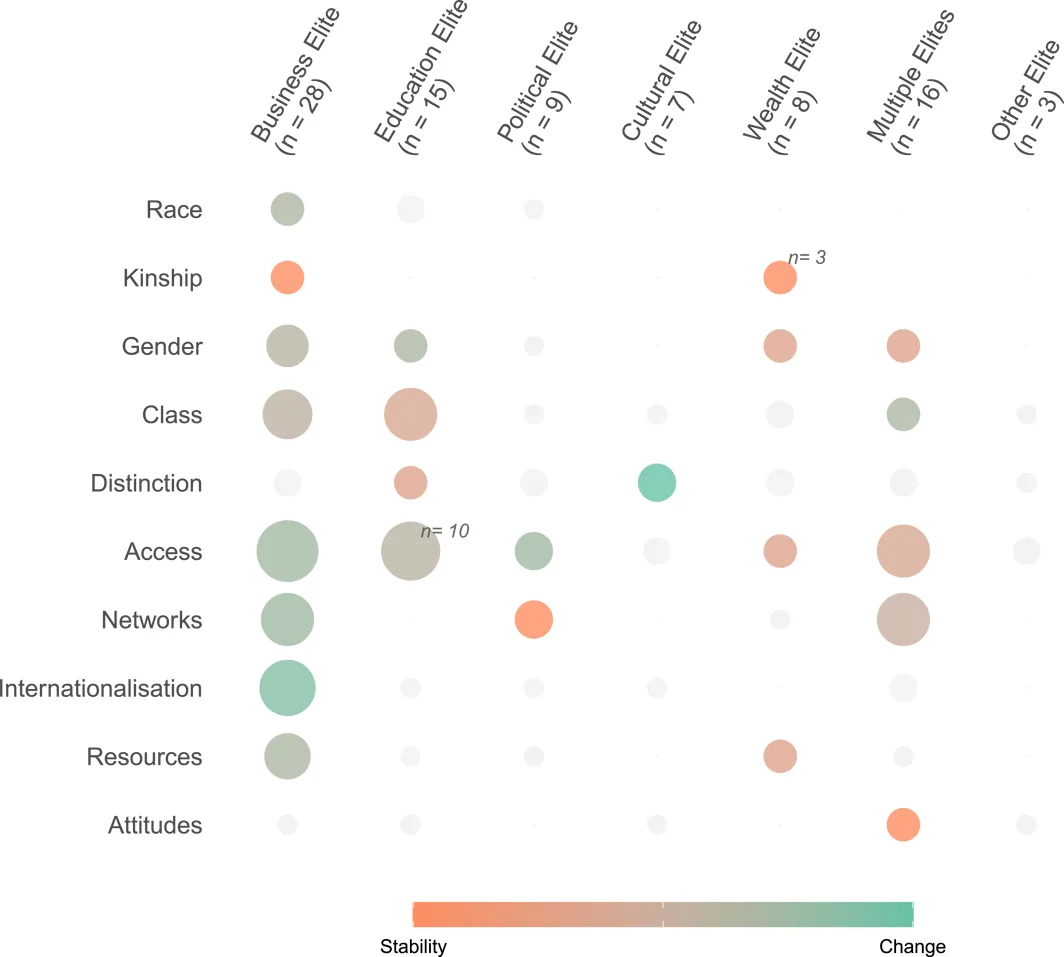
Temporal perspectives by elite types and research topic. The size of each circle in the matrix represents the number of articles for each hyphenated research area. Larger circles indicate a higher number of articles. The color of the circle reflects the proportion of articles focusing on change (green) versus stability (orange), shown only for cells with three or more articles. The classification of temporal focus was made based on the theory section of articles. Areas with less than three articles are left in gray. Note that while each article is assigned to a single elite type, it may be associated with multiple research topics (non‐exclusive coding). *Source:* Articles (*n* = 86) with a focus on change or stability *within* elites from the literature corpus on the sociology of elites.

To understand the patterns of temporal perspectives in studies on issues *within* elites, we examined whether certain types of elites, or certain research topics are more often associated with change or with stability. Figure 2 presents these patterns in a matrix. It shows the distribution of articles by hyphenated research areas, combining elite types and research topics. The matrix displays article counts in each area. Larger circles indicate more articles, while smaller circles indicate fewer articles. The color of each circle reflects the proportion of articles focusing on change (in green) versus stability (in orange).

A first observation from Figure 2 helps us to determine which areas receive most scholarly attention. Studies addressing Business Elites and Multiple Elites are particularly well represented. In contrast, Political Elites and Cultural Elites have received comparatively little attention. Among research topics, the theme of Access is most frequently addressed, while the topics of Race and Elites' Attitudes have received less scholarly attention. Hyphenated research areas with no articles in our sample appear as empty cells.

The main insight of Figure 2 is which research areas focus primarily on change within elites and which emphasize stability. The colors in the matrix indicate the dominant temporal focus of articles within each cell containing more than three studies. Orange cells reflect a primary emphasis on stability (meaning that the share of articles focusing on stability is high), green cells indicate a focus on change, and brown cells suggest that both temporal dimensions are addressed. In the following we outline the dominant foci in hyphenated research areas and highlight studies that are representative of the main patterns observed. The data show that studies on Wealth Elites and on Multiple Elites tend to emphasize stability. Research on Wealth Elites discusses, for example, strategies such as tax‐efficient philanthropy or legacy management (Sklair and Glucksberg 2021) and the role of family networks and intermarriage to maintain intergenerational wealth (S. O’Brien 2024). Analyses of gender relations within high income households also highlight stability, noting that work divisions remain largely unchanged due to enduring policy environments, ideal worker norms, and the effects of rising wealth concentration (Yavorsky et al. 2023). It is important to recall the scope of our review. As discussed in Appendix A2, our sample underrepresents research on income and wealth elites identified through terms such as “top earners” or “the 1%.” Including a broader set of keywords would therefore increase the size of the literature on this specific type of elite; however, the overall temporal patterns would likely remain similar, focusing on reproduction of privilege and concentration in resources. Cultural Elites, in contrast, are more often discussed as evolving (D. O’Brien and Ianni 2023; Pedersen et al. 2018). For example, a study on Beijing and Shanghai's elite discusses how economic reforms that promoted wealth accumulation, increasing exposure to Westernized education, and a Confucian tradition that places strong emphasis on self‐cultivation have shaped tastes of cultural elites (Li 2021).

Regarding research topics, we find that Nationality is most commonly approached through the lens of change. Studies examine themes such as global talent mobility among business elites, the influence of globalization and business friendly policies on elite recruitment (Beaverstock 2018), the growing importance of cosmopolitan capital (Bühlmann et al. 2013) and the increasing number and cohesion of transnational actors within the global corporate elite (Carroll 2009; Heemskerk 2011). In contrast, the diagram shows that studies on the topics of Kinship, or on Attitudes are more often framed through a lens of stability. To give an example, Teigen et al. (2023) examine elite attitudes toward boardroom diversity. The article argues that persistent gender inequality in top positions is closely tied to elites' reluctance to promote diversity among themselves.

While it is challenging to distill a general interpretation from the rather scattered literature, it is interesting to observe that social change seems to be related to elites' reactions to external pressures. In this light, elites appear less as architects of social change than as its reluctant curators, selectively opening the gates of change just wide enough to preserve their place within it.

### 4.2 Blurred Temporal Conceptions

Beyond the notable temporal patterns that we identify, a fundamental insight is that discussions of change and stability frequently appear together. As shown in Figure 2, even within specific combinations of elite types and research topics, authors adopt varied perspectives. For example, in the topic group on Gender or Class, some studies emphasize transformation, while others focus on continuity. These hyphenated research areas appear colored in brown.

In fact, contrasting temporal perspectives can coexist within single studies when authors discuss processes of change alongside mechanisms of stability. In our approach, we prioritized the dominant dimension, to be able to show distributions across research areas. A close reading of articles, however, match more complex conceptions of temporalities visible. For example, Ellersgaard and Larsen (2023) in a study on the power elite in Denmark, find that 55% of individuals in the elite core were newcomers between 2012 and 2017, which hints at strong evolutions. At the same time, they argue that organizational foundations of elite power remained highly stable. This indicates significant turnover at the personal level but continuity at the institutional level. Similarly, Gamsu (2018) demonstrates how elite schools in England strategically adopted traditional elite practices such as rugby, academic focus, and the prefect system to reposition themselves within the elite education landscape. At the core of the inquiry is thus one of adaptation and change. But at the same time, the author argues that the process of institutional “mimicry” reinforced cultural cohesion and enabled the schools to maintain their status, showing how change can actually serve to preserve elite continuity.

The strong interconnection between the themes of change and stability likely stems from several factors. One is that there is a certain ambiguity surrounding the question of what qualifies as meaningful or substantive change. Indeed, the definition of what constitutes change is far from consensual among authors. Another factor is that researchers may approach their work with certain theoretical expectations, only to encounter findings that point in a different direction. Some studies, for example, start off by addressing big markers of change, theorizing the impact of globalization or financialisation, only to empirically find that little evolves among the elite itself. Conversely, some studies set out to track changes in elites with extensive data, only to then fit the findings under a theoretical framework of persistence.

Overall, the literature discusses both how some elite groups adapt internally and how class backgrounds, and institutional arrangements in some elite groups help reproduce social hierarchies and exclude outsiders.

## 5 Social Change Driven or Impeded *By* Elites

A smaller strand of the reviewed literature focuses on the influence of elites, examining them as either agents of change or guardians of the status quo. In these articles, authors shift their focus beyond questions concerning elites themselves, centering instead on the role elites play in enabling or constraining processes of social change. These studies analyze elites' capacity to shape, direct, or obstruct transformation within various social fields. Within the Literature Corpus, 29 articles explicitly address how social change is driven or impeded *by* elites.

### 5.1 Guardians of the Status Quo

**FIGURE 3:**
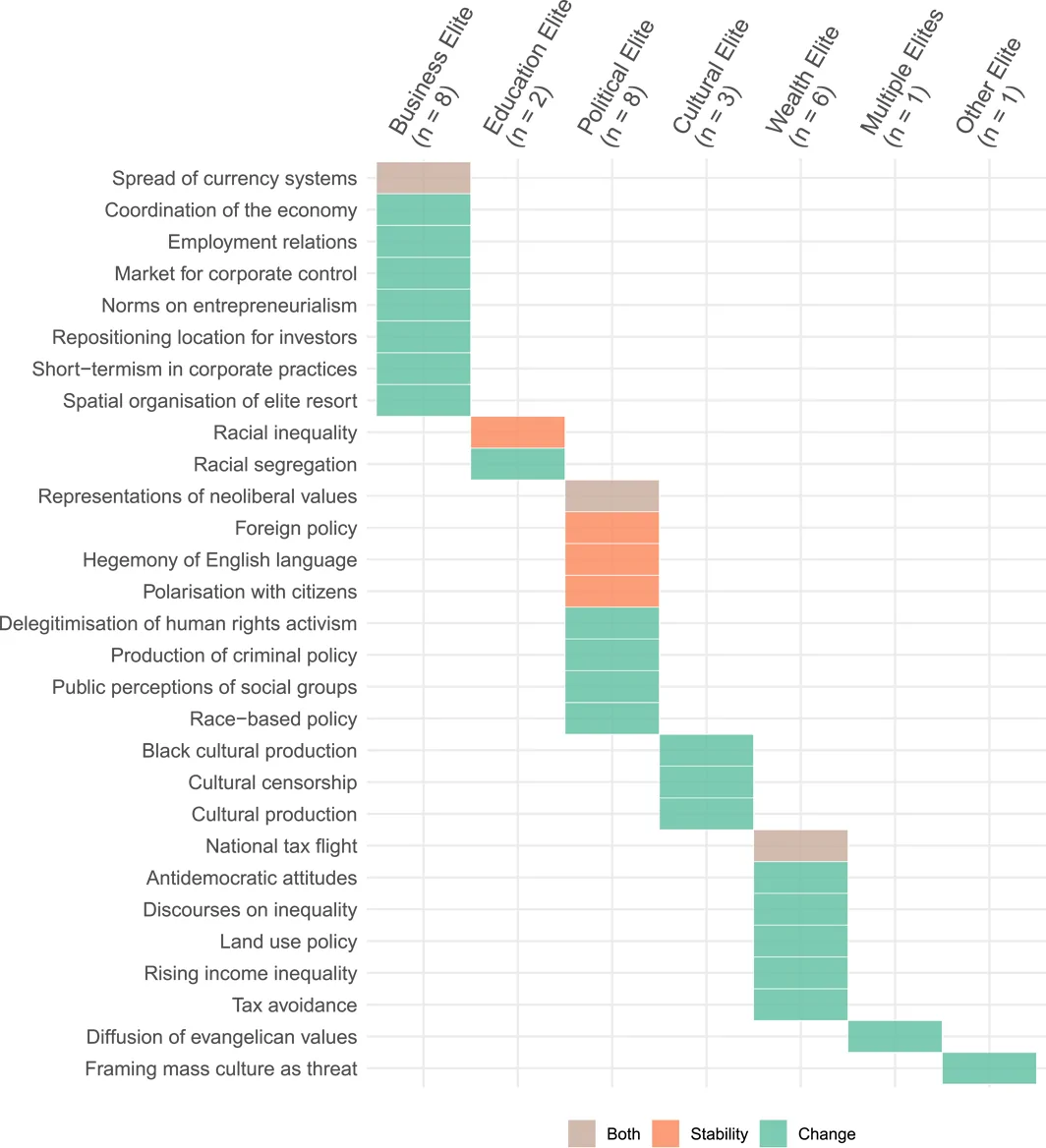
Temporal perspectives by elite types and influence spheres. Each line represents one article (total *n* = 29). The color of the rectangles reflects the proportion of articles focusing on stability (orange) versus change (green), or both (brown). The classification of temporal focus was made based on the theory section of articles. *Source:* Articles (*n* = 29) in the group of articles treating influence *by* elites from the literature corpus on the sociology of elites.

Overall, the contributions reviewed vary in their empirical focus and conceptual framing, yet they collectively underscore the importance of elites in broader social processes. As shown in Figure 3, relatively few studies focus on elites as guardians of the status quo (represented in orange). Among the various elite types examined, political elites are most strongly associated with stability. Scholars have documented how political elites impede change in domains such as foreign policy, perpetuate polarization between themselves and citizens, and sustain English language hegemony.

De Graaff and Van Apeldoorn (2021) describe the historical stability of the US foreign policy elite by documenting its enduring connections to corporate and policy‐planning networks across successive administrations. Davis (2015) discusses the role of UK political elites in maintaining a disconnection with citizens and their political representatives in mature democracies, arguing that this disconnection is characterized by a divergence between elite priorities and public concerns, ultimately perpetuating political apathy. Drewski (2024) examines how European Commission officials engage in symbolic struggles to define the legitimate forms of linguistic cultural capital. Privileging English as the vehicular language within the Commission is shown to relate to pre‐existing opportunity structures for language learning in officials' countries of origin, perpetuating unequal opportunities for participation within the transnational space of the Commission.

These studies suggest that elites can function as guardians of the prevailing social order through two primary mechanisms: by sustaining networks or maintaining positions of disembeddedness, and by upholding cultural standards that reinforce exclusionary norms and reproduce existing inequalities.

### 5.2 Agents of Change

Most studies examining elite influence portray elites as agents of change. This pattern is illustrated in green in Figure 3. The literature encompasses both conventional and less conventional spheres of elite influence. Common areas of scholarly inquiry include policy influence (De Graaff and Van Apeldoorn 2021; Duffy et al. 2010; Duxbury 2024), elites' influence on employment relations and economic coordination (Benton et al. 2021; Benton and Cobb 2019; Bühlmann et al. 2012; Widmer 2011) and their role as norm setters (Holmqvist 2024; Kantola and Kuusela 2019; Kuusela and Kantola 2023; Shriver et al. 2020). However, scholars have also explored less conventional domains of influence, such as the diffusion of evangelical values (Lindsay 2008), elite media ownership and cultural censorship (Rossman 2004), and the spatial transformation of elite resort destinations, such as the conversion of a coastal town into a space accessible almost exclusively to millionaires (Bruno and Salle 2018).

A closer examination of these studies reveals a first, somewhat counterintuitive, insight regarding how scholars conceptualize the effective degree of elites' intentionality. In fact, many studies characterize change as an unintended by‐product of shifting configurations rather than as the result of deliberate elite action. The outcomes of elite strategies may produce transformative consequences even when those consequences are not intentional. For example, firms' adoption of short‐term strategic behaviors has been linked to the (unintended) weakening of elite cohesion (Benton and Cobb 2019). In education, a study on elite‐driven school segregation in South Africa argues that segregation emerged not from explicit intent, but from the aggregation of individual choices made within families (Gruijters et al. 2024). By contrast, change is rarely identified as the direct effect of elite strategic and purposeful action. A notable exception concerns administrative elites in communist Czechoslovakia, who actively sought to defend their specific cultural capital by deliberately discrediting that of an oppositional activist movement (Shriver et al. 2020).

A second surprising insight is that studies which empirically investigate how elites drive change are rare. Indeed, many articles identify elite practices that are likely to generate social change, yet the implications of such change are merely alluded to or implied. Examples include elite appointments during the Trump administration (De Graaff and Van Apeldoorn 2021), shifts in investment patterns (Nau 2013), elite political disengagement (Davis 2015), symbolic performances by monarchies (Holmqvist 2024) and elite practices of tax avoidance (Shiffer‐Sebba and Harpaz 2024). All of these developments potentially carry significant large scale social transformations (e.g., on foreign policy, gender equality, social inequalities, and e.t.c.), but the transformations themselves are not the primary focus of the articles.

Fewer studies explicitly investigate social change as their central theoretical or empirical object. Authors who make explicit efforts to analyze the consequences of elite activities on broader social outcomes discuss, for instance, how cohesive corporate networks enhance benefits for white‐collar workers (Benton et al. 2021), how elite polarization is linked to shifts in criminal law production (Duxbury 2024), or how transformations among business elites (such as the rise of foreign managers) lead to fragmentation in corporate networks (Bühlmann et al. 2012). The scarcity of such studies may be attributable to disciplinary logics but also to data constraints. Indeed, the rare studies that provide empirical insights into elite‐driven social change need to adopt distinctive methodological approaches. Rather than focusing solely on elites themselves, they primarily utilize data that capture the social phenomena affected by elite actions. For example, to examine racial and social segregation in South African schools following the end of apartheid, Gruijters et al. (2024) draw on two nationwide school surveys. Some studies incorporate data on both elite dynamics and the broader social outcomes they influence. Benton and Cobb (2019), for instance, employ five distinct datasets to document the fragmentation of the corporate elite (treated as the independent variable) and to assess its impact on the adoption of short‐termist corporate strategies (the dependent variable).

In summary, the literature examining how elites drive or impede social change spans a broad range of spheres of influence. Notably absent, however, are studies that empirically investigate elite‐driven transformations in cultural practices and tastes. Consequently, the literature offers limited insight into the broader cultural ramifications of elite behavior. Studies focusing on elites as agents of change, tend to portray them as unintentional agents. Broader social transformations are typically discussed as incidental outcomes of elite behavior or as emergent effects of individual strategies rather than as the products of coordinated collective action.

## 6 Pathways for the Study of Elites

### 6.1 Reversing the Time Lense

As in any field, blind spots remain. The matrix on change and stability within elites helps identifying these gaps in the collective understanding of elites as a sociological phenomenon. We show that several important themes concerning elites receive surprisingly little attention in the leading sociology journals we have analyzed. These include topics such as religion, sexuality and migration, and concern certain elite types, notably military or law elites. Similarly, we point out hyphenated research areas which remain understudied, for example “Race ‐ Cultural Elites”; “Nationality ‐ Wealth Elites” or “Kinship—Political Elites”.

What the matrix of change and stability reveals, beyond gaps linked to particular elite types and topics, is the potential of generating new questions by inverting the conventional temporal lens. Questions about wealth and income, for example, are often approached through a prism of stability. Reversing the perspective can lead to new questions. For instance, which social configurations contribute to elite decay, that is, the loss of wealth? At what point does wealth concentration among a few generate conflicts within elites or tensions with society at large? Nationality or internationalization of elites, by contrast, are usually studied through a prism of change. Reversing the perspective here opens up a different set of inquiries: Which elite subgroups remain predominantly national in orientation? What mechanisms are employed to exclude or limit access by international challengers? Engaging the matrix from its inverse side—reversing both time and focus—reveals a range of underexplored questions for elite research.

While researchers often frame their interest in elites around questions of power, our review shows a clear focus on issues that concern elites themselves. Building on this predominant focus, research could ask: What do changes within elites imply for broader social change? Few scholars have empirically addressed these connections. A rare exception is Reeves and Friedman (2024) book, where the authors use vignette experiments to test whether compositional shifts within elite groups influence policy preferences and decision‐making processes. Elite sociology has developed strong foundations in analyzing actors' networks, career trajectories, educational backgrounds, socio‐demographic traits, and elite attitudes or discourses. The challenge in studying power of elites lies in mobilizing this knowledge and use it to investigate how elite characteristics and configurations enable or sustain various forms of influence and opportunity hoarding (Gilbert and Sklair 2018). A promising direction for future research is thus to systematically link shifts within elites to their broader effects on social change.

### 6.2 Situating Observations on a Temporal Spectrum

Our review shows that temporal concepts can blend into each other. Empirical findings are often difficult to place clearly along a continuum between relative stability and drastic transformation. This raises a fundamental question for elite research: what qualifies as a meaningful degree of change? Put differently, how substantial must a shift be to seen as genuine transformation? A core strength of elite research lies in its empirical diversity in that scholars study various actors of power across a wide range of social, political, and historical contexts. Standardiing definitions of temporal change would risk flattening this plurality. Instead, we suggest that future research can move toward greater precision not by imposing uniform definitions, but by adopting more refined concepts of change and adopting transparent strategies for comparison.

The first step in this direction is to develop more refined ways of conceptualizing temporal dynamics. Dominant concepts tend to circle around notions of increase and decline, often expressed in different guises such as rise and fall, or ascent and descent. There are, importantly, highly intriguing examples in the elite literature that move beyond linear understandings of change. These include notions of “back to the future,” “reconfigurations of the past,” and debates about “neo‐patrimonialism,” as well as ideas like “the invention of tradition”. Building on this, we argue that elite sociology could explore further how stability and change may coexist or interact in unexpected ways. Processes can be multilinear, and marked by alternative trajectories, or substituting other processes (Sztompka 1994). Or they can be non‐linear, marked by breakthroughs when passing a threshold for example (Sztompka 1994). Concepts such as tipping points, ruptures, fluctuations, cycles, rhythms, delayed effects, and cascading impacts offer useful tools for capturing the layered nature of social transformations. Expanding the concepts around time and change would allow elite scholars to embrace more fine‐grained temporal approaches to understanding how elites respond to, shape, and are shaped by broader social change.

A second step in this direction is to adopt multiple and transparent strategies for comparison of observed change. A common comparison category in elite research and sociology more broadly is the comparison across space; for example, following the evolution of a phenomenon across different national contexts (or other spatial entities, e.g., cities). For *within* elite research empirical findings can be further contextualized through comparisons across elite types. For instance, to assess whether social closure in access to business elites has changed minimally, or modestly, over the past century, the comparison with elite groups in public administration or law provides valuable insights (see e.g., Hartmann (2025)). A less frequently activated dimension of comparison involves making normative assumptions or prioritizations explicit. For example, if researchers adopt a normative commitment to gender equality, the continued underrepresentation of women in elite positions may be interpreted as a sign of slow or insufficient change. Against the assumption of gender equality, gradual increases in female representation may be judged as maintenance of stability if change fails to transform underlying of influence. The same applies to the underlying assumptions about which aspects of elite dominance are considered most important. Similar considerations apply to assumptions about which aspects of elite dominance are regarded as most significant. If, for instance, social class is treated as the decisive factor, making this assumption explicit helps us see continuity in a different light: apparent turnover among elites may conceal enduring patterns of privilege, as new entrants continue to be drawn from the same social strata. In this sense, the idea that “everything needs to change for everything to remain the same” (François and Lemercier 2016) acquires a distinct meaning.

To situate observations on the continuum of change and to qualify what counts as meaningful transformation, elite sociology could benefit from adopting more fine‐grained conceptions of change and more transparent strategies of comparison. This means placing temporal observations within multiple reference points, since any observed change, whether minor or substantial, is always relative.

### 6.3 Addressing Intentionality in Agents of Change

A striking finding of our review is that, in most cases, scholars analyzing forms of change by elites focus on instances where transformations are not deliberate and strategic efforts to reshape society to elites’ advantage but as unintended outcomes. Elites are thus often portrayed as unintentional agents of change in sociological studies. Social, economic, political, and cultural shifts beyond elites' immediate control create new constraints and opportunities that compel them to adapt. These external pressures may arise from technological innovation (e.g., the diffusion of artificial intelligence), climate change, demographic transitions, social movements, or political realignments, among many other sources. In response, elites modify their practices in ways that are typically defensive, adjusting their behaviors to safeguard their power in the face of perceived threats. Political elites, for instance, may co‐opt the language of social movements to blunt dissent, or corporate elites may adopt symbolic reforms to pre‐empt regulation. In turn, such adaptations may generate unintended externalities that can have broader transformative consequences. For example, corporate elites adopting diversity rhetoric may unintentionally reshape collective understandings of fairness and merit, while media elites modifying coverage to retain audiences may inadvertently shift the boundaries of public debate on issues like migration, gender, or the environment. This phenomenon is manifestly worthy of study given the structural effects it may generate, even when those consequences remain unintended.

However, this observation also reveals significant potential for future research. First, a productive way forward is to re‐emphasize intentionality in elite studies. Doing so would allow scholars to identify instances of change arising from elites' purposive interventions in shaping societies to their advantage. For instance, financial elites purposively developed the subprime mortgage market in the 2000s, expanding credit access while simultaneously creating new avenues for profit. Beyond the economic sphere, cultural elites may deliberately reconfigure educational curricula or cultural institutions to entrench particular worldviews, or policy elites may strategically redesign welfare systems to redefine citizenship and social belonging. In a contrasting perspective, Sherman (2021) suggests that elites may sometimes act against their own interests, intentionally shaping institutions or policies in ways that disadvantage their own capital accumulation, thereby functioning as “class traitors”. Understanding these dynamics requires attention not only to the constraints elites face but also to the ambitions and strategies through which elites seek to transform social structures.

Secondly, our review exposes another blind spot in recent literature: elites are seldom theorized as guardians of the status quo. Social transformation can take many forms (from technological breakthroughs and demographic realignments to institutional reform and grassroots mobilizations), and elites have the power to play a decisive role in determining which of these developments advance or stall. When confronted with changes that threaten their position, elites may intervene directly to preserve existing hierarchies, blocking reforms or redirecting transitions in ways that protect established interests. Greater attention to elites as guardians of the status quo would enrich our understanding of how the boundaries of social transformation are drawn and defended. By examining elites both as strategic agents who intentionally remake social orders and as guardians who stabilize and defend them, future research can offer a more comprehensive account of how power is exercised, reproduced, and reconfigured in contemporary societies.

### 6.4 Anticipating the Future

Our review shows that scholars discuss elites in relation to the past and the present emphasizing their role in preserving structures as gatekeepers and in responding to pressures as agents. Social change relates to three dimensions, however, the past, the present and the future (Sztompka 1994). Beyond the intentionality of elite actors, the sociology of elites could move forward by shedding light on how elites formulate projects of the future. This shifts attention from viewing elites merely as respondents to external forces to understanding them as *proactive* shapers of what is to come.

We know relatively little about elite's relationship with the future, on forward‐looking perspectives in elite decision‐making and strategic positioning. Future‐making is a central axis of power accumulation since it both can shape elite actors' personal trajectories, national or transnational capital building (Maxwell and Lillie 2024) and reshape social and material landscapes (Bolzoni et al. 2025). This future‐oriented capacity can be understood in three interrelated ways. First, aspiration, where elites pursue desired futures shaped by their dispositions and the opportunities available to them. Second, anticipation, in which elites make present decisions strategically to navigate toward uncertain futures. Third, fictional expectations, where imaginative projections of possible futures guide actions under conditions of uncertainty.

Scholars have started to observe these forms of future‐making in elites' responses to recent global disruptions. During the most recent global pandemic, elite actors put forward anticipatory strategies by relocating to private chalets in the Alps, seeking refuge from contamination and social measures (Bolzoni et al. 2025). Similar anticipatory strategies could be seen in the investments in climate bunkers by the super‐rich to safeguard themselves from climate catastrophes (Cousin and Schultz 2024). As such, exploring elites as architects of the future allows to investigate how these elite practices actively shape the distribution of security, mobility and resources due to elites' disproportionate access to and control of different forms of capital.

This gap raises an important and underdeveloped question in elite research: What do elites want? By integrating future‐oriented perspectives into elite studies, scholars can move beyond models of elite reproduction and develop a more comprehensive understanding of how elites actively shape long‐term social transformations. Tackling these questions might become particularly relevant in a context of ongoing struggles over the role of democracy in contemporary societies and signs of a new global order. While contemporary research is situated in a different scientific and geopolitical context than early elite scholarship, we might go back to the future. As a new generation of elite scholars, we call for clarity and rigor in anticipating the challenges and possibilities ahead.

## Funding

This project has received funding from the Swiss National Science Foundation (grant agreement number 215001).

## Conflicts of Interest

The authors declare no conflicts of interest.

## Figures and Tables

**FIGURE A1:**
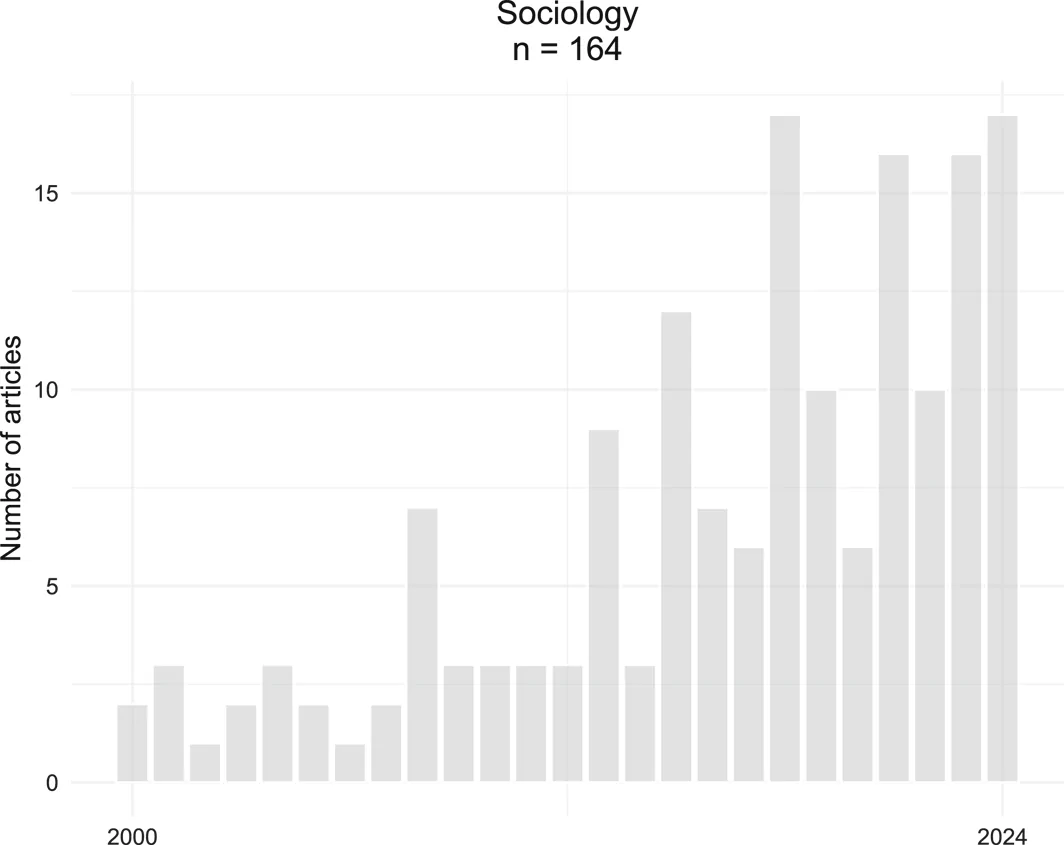
Number of publications in elite sociology over time. Articles from the literature corpus.

**FIGURE A2:**
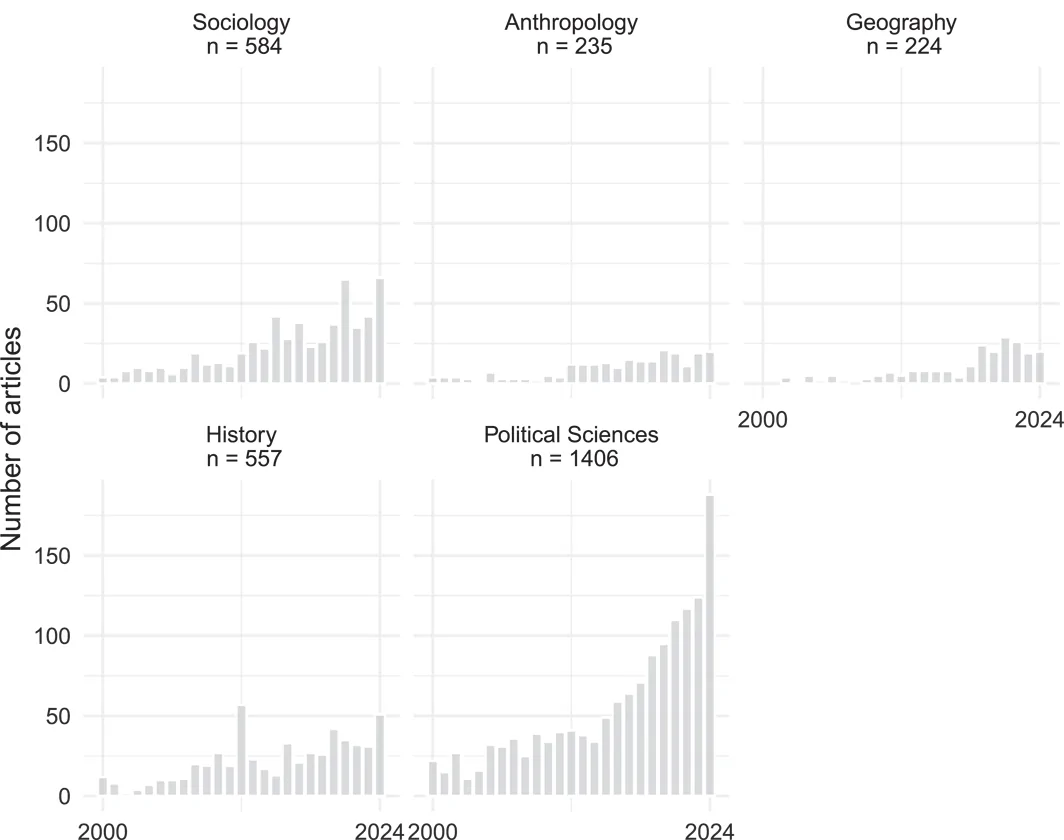
Number of publications in broader elite studies over time. Articles from the Web of Science.

## Data Availability

The data supporting the findings of this study are available from the corresponding author upon reasonable request.

## Appendix A

### A1 Author Collective

A particularity of our approach is that we worked as a collective of new generation researchers. Most of us met at the 2024 Elite Summer School in Lausanne. Our collective strategy involved defined responsibilities, shared goals, and regular meetings. We agreed on authorship conditions from the beginning, which set a minimum contribution for every member of the collective. The main writing responsibility was with the two leading authors, but the process was iterative and included extensive feedback sessions. The collective contributed ideas to each part of the article and took part in decisions about what to emphasize and what to leave out. Within our collective, we aimed to cultivate a culture that encouraged open exchange of ideas and thorough discussion. This helped us broaden our perspective on elite sociology and social change and to formulate new ideas for the future direction of the field.

### A2 Literature Corpus: Sample and Approach

Using the terms “elite” or “elites” in the title represents a rather narrow sampling choice. To explore which areas of elite research might appear under alternative terminology, we cross‐checked a list of related terms including for example “super‐wealthy”, “rich”, “field of power” or “CEO”. We identified all articles containing any of these terms in their abstracts from three randomly selected journals: the American Sociological Review, the Socio‐Economic Review and Global Networks. We then assessed each article manually to determine its relevance to the sociology of elites. Table A1 shows the share of false positive articles for each term. A high share of false positives indicates that most articles with a given term are not engaging with elite sociological interrogations. This is particularly the case for articles with the terms “rich” (98.6%), “wealth” (80.4%) or “wealthy” (75.8%). A lower percentage of false positives indicates a higher alignment with actual elite studies. Table A1 shows that many articles on income (affiliated with the terms “top earners”, “super‐rich”, “1%”) contribute to the sociology of elites without explicitly using the term “elite.” It should thus be noted that our sampling approach likely underestimates the significance of work on income elites.

**TABLE A1:** Proportion of false positive articles identified using elite‐related terms. A lower percentage of false positives indicates a higher alignment with actual elite studies.

Keyword	% False positives
Super‐wealthy	—
Rich	89.6%
Wealth	80.4%
Wealthy	75.8%
Field of power	50.0%
CEO	37.5%
Upper class	33.3%
Inner circle	0.0%
One percent	0.0%
Super‐rich	0.0%
Top earners	0.0%
Top income earners	0.0%

### A3 Literature Corpus: List of Journals

To constitute the Elite Sociology Literature Corpus, we defined a subset of leading sociology journals. Public rankings proved unsuitable for our purposes because of issues with subfield prioritization. For example, the OOIR ranking included journals irrelevant to elite sociology, such as *Annals of Tourism Research*, while SCImago combines sociology and political science journals in a single category. We therefore relied on a reputational approach. We worked with the Dan Hirschman Ranking (2017 and 2020), a crowdsourced ranking of generalist and specialist sociology journals published on scatterplot, which seems best suited to reflect broad reputational hierarchies in the broad field of sociology. We then refined the list by reprioritizing journals in consultation with established scholars in elite sociology (in 2024). Journal hierarchies are organized around clusters and specialist journals can have a low impact factor, but a high influence in certain subdisciplines. Although this step draws on contemporary assessments, the resulting prioritization is unlikely to differ meaningfully from earlier periods given the long‐term stability of elite journal hierarchies in sociology. Our process ensured that the journals included in our sample are central and leading outlets for elite sociology (Table A2).

**TABLE A2:** Journals included in the literature corpus.

	*n* articles	%
Acta sociologica	8	4.9
American journal of sociology	5	3.0
American sociological review	9	5.5
Cultural sociology	6	3.7
Economy and society	3	1.8
European sociological review	6	3.7
Focaal	3	1.8
Global networks	19	11.6
Poetics	12	7.3
Social currents	2	1.2
Social forces	12	7.3
Social problems	4	2.4
Social science research	3	1.8
Socio‐economic review	13	7.9
Sociological science	0	0.0
Sociology	15	9.1
Sociology of education	5	3.0
Socius	5	3.0
The british journal of sociology	14	8.5
The sociological review	20	12.2
Total	164	100

*Note:* The column *n* articles indicates the number of articles that appeared in each journal between 2000 and 2024 with elite/s in the title. The column % indicates the share of all articles of a given journal in our elite sociology corpus.

### A4 Rise of Publications in Elite Sociology—Literature Corpus

Figure A1.

### A5 Rise of Publications in Broader Elite Studies—Web of Science Corpus

To assess whether the observed increase in elite studies reflects a growing concentration of such publications within our selected set of key journals, we analyzed trends in a larger, extended sample of sociology articles drawn from the Web of Science Core Collection (WoS), a comprehensive database of scholarly publications. We downloaded the data on 19.05.2025. We identified 3006 social science articles published between 2000 and 2024 in the Web of Science Database with indications on publication year and discipline, all of which include the term elite/s in the title. We code research areas as sociology, political sciences (including WoS Categories “political sciences”; “international relations”), history, anthropology (including WoS Categories “anthropology”; “ethnic studies”; “cultural studies”) and geography (including WoS Categories “geography” and “environmental sciences”). Articles on sports elites were excluded. We excluded books and book chapters and selected exclusively journal articles to constitute the corpus, as we wanted to restrict the analysis to peer reviewed research. Note that the sample of literature in the sociology of elites in the Web of Science is larger than in our Literature Corpus, because it includes publications beyond the key 20 journals. The graph shows a broader pattern of increasing numbers of elite studies across disciplines over time (Figure A2).
